# Attitude, practice and its associated factors towards Diabetes complications among type 2 diabetic patients at Addis Zemen District hospital, Northwest Ethiopia

**DOI:** 10.1186/s12889-020-08953-6

**Published:** 2020-05-26

**Authors:** Yitayeh Belsti, Yonas Akalu, Yaregal Animut

**Affiliations:** 1 Department of Physiology, College of Medicine and Health Sciences, University of Gondar, Gondar, Ethiopia; 2 Department of Epidemiology and Biostatistics, Institute of Public Health, College of Medicine and Health Sciences, University of Gondar, Gondar, Ethiopia

**Keywords:** Attitudes, Practice, Diabetes mellitus, Complications, Associated factors, Ethiopia

## Abstract

**Background:**

This study aimed to assess the level of attitude, practices, and its associated factors towards complications of diabetes mellitus among type 2 diabetes patients.

**Methods:**

An institution-based cross-sectional study was done on type 2 diabetes patients coming to the diabetes outpatient department at Addis Zemen District Hospital in Northwest Ethiopia. Interviewer-administered structured questionnaires were used to collect data from 402 patients. Multivariable logistic regression was employed to decide on factors related to practices and attitudes towards diabetes complications. AOR with 95% CI and *p*-value under 0.05 was considered to select significantly associated variables.

**Results:**

Two-thirds of the study participants (65.2% (95% CI: 60.2, 69.4)) had a good attitude level while less than half of study participants (48.8% (95% CI: 44.0, 53.5)) had a good practice on diabetes complications. Educational status of read and write (AOR = 2.32, 95% CI(1.26, 4.27)), primary school (AOR = 4.31, 95% CI(2.06, 9.02)), high school and above (AOR = 2.79, 95% CI (1.41, 5.50)), and urban residence (AOR = 1.80, 95% CI (1.12 2.91)) were significant factors for good attitude while educational status of read and write (AOR = 1.96, 95% CI (1.06, 3.61)), and high school and above (AOR = 2.57, 95% CI (1.32, 5.02)) were associated with diabetes complication practices.

**Conclusions:**

A greater proportion of diabetes patients had a relatively good attitude but poor practice towards diabetes complication preventions. Residence was a contributing variable for a good attitude while the level of education was significantly associated with both practice and attitude. The current study suggests the need for structured educational programs about diabetes complications regularly to improve patient’s attitudes and practice.

## Background

Diabetes mellitus (DM) is a serious, chronic disease that occurs either by impaired insulin secretion or insulin resistance or both [1]. DM is one of the four priority non-communicable diseases (NCDs) targeted for prevention and control by the World Health Organization 2011 [2]. It was estimated that in 2019 there were 500 million people with diabetes worldwide [3] and predicted to be 693 million by 2045 [4]. Type 2 diabetes will be the predominant public health problem in Africa and expected to be 28 million by 2030 [5] and 41.6 million in 2045 [6]. In Ethiopia, one of the top five African countries regarding the prevalence of diabetes in the age range of 18–99 years, there were 2,652,129 cases of diabetes in 2017 [6]. Studies in various parts of Ethiopia showed that the prevalence of diabetes varies from 0.3 to 7.0% [2].

Diabetes is identified as the main cause of premature death and disability [2, 6, 7]. Most of the death and disabilities are caused by diabetic complications that damage the heart, blood vessels, eyes, kidneys, and nerves [8, 9]. Such damage increases the chances of foot ulcers, infection, and the eventual need for limb amputation [10]. Diabetic retinopathy, one of the main diabetic complications, is an important cause of blindness. Diabetes is also among the leading causes of kidney failure [7]. According to the WHO report in 2016, DM is attributed to 2% death among all causes of death in Ethiopia which is mainly due to its complications [7]. Studies conducted on the prevalence of acute and chronic complications in different parts of Ethiopia showed that the prevalence of retinopathy 5.0 to 43.4%, neuropathy 6.0 to 41.0%, foot ulcer 1.7 to 17%, nephropathy 1–20%, hypertension 3 to 39%, and erectile dysfunction 1 to 22% [11].

The International Diabetic Federation (IDF) estimated that the total health expenditure due to diabetes in 2017 was $3.3 billion worldwide. Most nations spend 5to 20% of their health budget on diabetes, predominantly to prevent and treat diabetic complications [6]. All major complications of diabetes are preventable by good control of blood glucose levels, blood pressure, and cholesterol levels. This requires a high level of education of the person with diabetes in managing their condition, access to insulin, oral medications, and monitoring equipment [12].

There is an increasing amount of evidence that patient education is the most effective way to lessen the complications of diabetes and its management by improving their attitude and practice [13]. Unfavorable attitudes and psychological problems like depression are common among diabetes patients and can lead to poor diabetes care provoking diabetic complications [14, 15]. There is powerful evidence that individuals who are educated and diligent in their diabetes self-care achieve better and durable diabetic control [16, 17]. Past studies on knowledge, attitude, and practice towards the prevention of diabetes complications consistently revealed the requirements of better awareness on prevention, diagnosis, and risk factor control of diabetes [18]. A good attitude towards DM complications helps patients to change any harmful dietary and lifestyle habits [19].

Though there are few works of literature on the prevalence of diabetic complications, there is no published data on attitude and practices towards complications of diabetes among type 2 diabetic patients in Ethiopia. Thus, this study was conducted to determine the attitude and practices and its associated factors towards diabetic complications among patients at Addis Zemen District Hospital, Northwest Ethiopia. The information generated from this study will provide important input for designing diabetes complications prevention strategies, which help to improve the quality of care for type 2 diabetic patients and to reduce the burden associated with diabetes complications.

## Methods

### Study setting, design, and period

We conducted an institution-based cross-sectional study from April 02, 2019 to June 02, 2019 at Addis Zemen District Hospital, South Gondar, northwest Ethiopia. Addis Zemen District Hospital is found in Addis Zemen town, which is a central town of Libo Kemkem District, South Gondar Zone, Amhara regional state. It is found 90 km (km) away from Bahir Dar (the capital city of Amhara Regional State) and 656 km northwest of Addis Ababa.

### Study population and eligibility criteria

Type 2 diabetic patients who were present at the diabetic center during the study period were incorporated with the exception of those individuals who were in an extreme disease condition that restricts them to respond to questionnaires and the individuals who were health professionals.

### Sample size determination, and sampling technique

The sample size was estimated using a single population proportion formula considering *p* = 50% (magnitude of good practice towards DM complications), a 95% degree of certainty, and a 5% margin of error. Accordingly, the sample size became 384. After including a 5% non-response rate, the final sample size was 404. Systematic random sampling technique was employed to select the study participants. During the study period, there were 1144(N) type 2 diabetic patients on follow up, listed on the diabetic registration chart with their respective medical registration number. Then we determined the number of sampling intervals K by dividing 1144(N) by the required sample size 404(n), which became 3(K). Then we selected the random start (the first sampling unit) from the first interval in the frame with simple random sampling. Then every 3rd unit samples were selected.

### Data collection instrument and procedure

An organized pretested interviewer-administered questionnaire was utilized to collect data regarding attitude, practice, and associated factors. The questionnaire was adapted from relevant literature [20–23] and contains 9 attitudes and 13 practice-related questions towards diabetes complications It also incorporates main socio-demographic factors like sex, age, residence, occupation, marital status, educational status, income, duration since diagnosis as diabetic, and family history of DM. The questionnaire was prepared in English by language expert, and translated to local language (Amharic) and then translated back to English by another language expert to ensure its consistency and wording. Data were collected by five BSc nurses. From socio-demographic variables, educational level was categorized as follows; “cannot read and write”, “informal education and can read and write”, “primary school and can read and write”, and “secondary school and above”. The essential point here was to distinguish whether they can read and write not to miss the beneficial outcome of reading and writing on their attitude and practice. If the study participants attended primary school but couldn’t read and write we will put them under unable to read and write. In Ethiopia, some people attend informal and religious educations but not primary school and are able to read and write. Hence, we grouped such candidates under the category of read and write. Settling in the field outside of huge urban communities or towns in Ethiopia is alluded to as provincial inhabitants [24].

### Assessment of attitude

We had used 9 questions adapted from different literatures [25, 26] to assess the attitude of the patients. Each question had three choices of responses (agree, neither agree nor disagree, and disagree). A score of “1” was given for a favorable attitude (correct answer) and “0” for unfavorable attitude (incorrect answer) for each respective attitude questions [20]. All possible correct answers were summated out of 9. The diabetic patient’s attitude level towards diabetic complications was determined by adding the right answers and calculating the mean value as 5 with possible maximum correct answer of 9. Participants mentioning under the mean score of 5 correct answers grouped as having poor attitudes. The participants who mentioned five or more correct answers rated as a good attitude. Attitude questions and their respective response scores are presented in the attached Additional files.

### Assessment of practice

There were 13 inquiries that survey the practice of the patients about diabetic complications, 10 inquiries had a “Yes”, or “No” responses, “Yes” demonstrates the practice of suggested action. A score “1” was given for “Yes”, and “0” for “No”. The remaining three measures of practice questions scored as follows: How often have you done physical work or exercise in the last week? Those who respond “Never” was given a score of “0” but the remaining recommended options (daily, almost daily, 2–3 times per day and once a week) were given a score of “1”. For questions on non-recommended activities/habits like “How often do you drink alcohol?” the response never was given a value of “1” which is a suggested practice for diabetic patients while the rest of the responses (rarely, nearly every day, 2–3 times/day, and weekly) given a score of “0”. Finally, since the recommended type water for foot care is warm water but not too hot or too cold, for the question “What type of water do you use to wash your feet?” the response of warm but not hot water was recorded as “1” and cold water recorded as “0”. Then, the patient’s practice level was calculated by adding their responses and the maximum possible score was 13. A score of 8 and above were categorized as good practice, while the remaining as a poor level of practice [20, 27, 28]. Practice questions and their respective response score are presented in the attached Additional files.

### Data quality management/control

One day training was given for data collectors and supervisors about the data collection procedure and ethical issues. The pretest was conducted on 60 type 2 DM patients at the University of Gondar specialized Hospital DM clinic. Data collection was closely supervised by two health officer supervisors and investigators. The collected data were checked for completeness at a daily meeting. The data were cleaned and checked before analysis.

### Data processing and analysis

The data were checked for completeness and entered into Epi Info variant 7 and were traded to SPSS Version 20. Graphic measurements, for example, frequencies and rates were utilized. Bivariate analysis was done for every single autonomous variable with the outcome variables and factors with *p*-value0.2 and below were entered into a multivariable logistic regression model to recognize the independent associated factors of attitude and practice. Adjusted odds ratio (AOR) with 95% CI and p-value of less than 0.05 were considered to declare significantly associated factors.

## Results

### Socio-demographic characteristics

Among of 404 participants 402 responded well, giving a response rate of 99.5%. Of all respondents, 225 (56%) were female and 243 (60.4%) were urban inhabitants. Most of the respondents (150 (37.3%)) were aged 45–70 years. The majority of (304 (75.6%)) of the participants were Orthodox Christians and 348 (86.6%) were from the Amhara ethnic group. Of all participants, 118(29.4%) had a family history of DM. Out of the total respondents, 242 (61.3%) were married, 110(27.4%) were unable to read and write, were urban residents 85 (21%) were farmers, and 156 (38.8%) had a monthly income below 500 Ethiopian birr (Table 1).

**Table 1:** Socio demographic characteristics of Type 2 DM patients at Addis Zemen District Hospital, Gondar, Ethiopia, 2019(*N* = 402)

Variables	Frequency	Percent (%)
Sex		
Male	177	44.0
Female	225	56.0
Age (in years)		
18–30	134	33.3
31–45	118	29.4
45–70	150	37.3
Level of Education		
Cannot read and write	110	27.4
Informal education can read and write	124	30.8
Primary school and can read and write	77	19.2
Secondary school and above	91	22.6
Marital status		
Married	202	50.2
Divorced	37	9.2
Widowed	49	12.2
Single	114	28.4
Occupation		
Farmer	85	21.1
Government worker	46	11.4
Merchant	104	25.9
Housewife	77	19.2
NGO worker	90	22.4
Religion		
Orthodox	304	75.6
Muslim	68	16.9
Protestant	30	7.5
Ethnicity		
Amhara	348	86.6
Kimant	35	8.7
Tigrie	19	4.7
Residence		
Rural	159	39.6
Urban	243	60.4
Duration of DM (in years)		
[1–5]	270	67.2
[6–10]	93	23.1
>10	39	9.7
Type of medication they use		
Oral	133	33.1
Injectable	218	54.2
Both	51	12.7
Family history of DM		
Yes	118	29.4
No	284	70.6
Income(ETB)		
<500	156	38.8
500–1500	68	16.9
1501–2500	86	21.4
>2500	92	22.9

*Abbreviations: ETB = Ethiopian Birr, DM = Diabetes mellitus, NGO = Non-governmental organizations*

### Attitude of type 2 diabetic patients towards complications of Diabetes

About two-thirds of respondents (65.2% (95% CI: 60.2, 69.4)) had good attitude levels. Most (74.1%) of the respondents agreed that diabetic complications could be prevented. Nearly three quarters (74.1%) of respondents thought they could lead a normal life if they take appropriate measures for diabetes. Over two-thirds of the participants 69(16.9%) believed that diabetic complications can be prevented by having good glycemic control. The majority (79.9%) of participants agreed that regular exercise can prevent complications of diabetes. Dietary modification and weight reduction were considered important to prevent diabetic complications by 91.8% (*n* = 369) and 46.3% (*n* = 186) respondents, respectively. One hundred thirty-five (33.6%) respondents believed that their diabetic diet spoils their social life. Furthermore, 214(53.2%) respondents thought diabetics were the worst thing that ever happened to them (Table 2).

**Table 2:** Frequency distribution of participant’s response on attitude related questions on DM complications among Type 2 adult DM patients at Addis Zemen District Hospital, Gondar, Ethiopia, April, 2019(N = 402)

Variables	Frequencies	%
I can lead a normal life if I take appropriate measures for diabetes		
Agree	298	74.1
Disagree	64	15.9
Neither nor disagree	70	10
Regular exercise prevents further complication		
Agree	321	79.9
Disagree	25	6.2
Neither nor disagree	56	13.9
Glycemic control has no role in preventing complications		
Agree	68	16.9
Disagree	333	82.8
Neither nor disagree	1	2
Diabetic spoils my social life		
Agree	135	33.6
Disagree	232	57.7
Neither nor disagree	35	8.7
I could prevent diabetic complications		
Agree	298	74.1
Disagree	40	10
Neither nor disagree	64	15.9
Dietary modification is important to prevent diabetic complication		
Agree	369	91.8
Disagree	29	7.2
Neither nor disagree	4	1
Weight reduction is important to prevent diabetic complication		
Agree	186	46.3
Disagree	104	25.9
Neither nor disagree	112	27.9
I don’t tell people as I have diabetics		
Agree	38	9.5
Disagree	349	86.8
Neither nor disagree	15	3.7
Diabetes is the worst thing that have ever happened to me		
Agree	214	53.2
Disagree	173	43
Neither nor disagree	15	3.7
Total attitude level		
Good attitude	262	65.2
Poor attitude	140	34.8

Abbreviations: DM = Diabetes Mellitus

### Factors associated with attitude towards complications of Diabetes

The residence and educational status of the respondents had a significant influence on attitude. Subjects with educational level of informal school and can read and write were 2.3 times (AOR = 2.32, 95% CI (1.26, 4.27)) more likely to have a good attitude as compared to those who cannot read and write. Type 2 diabetic patients who attained primary school and can read and write were 4 times (AOR = 4.31, 95% CI (2.06, 9.02)) more likely to have a good attitude than those who cannot read and write. Similarly, those who conquered secondary school and above were 2.8 times (AOR = 2.79, 95% CI (1.41, 5.50)) more likely to have a good attitude than those who cannot read and write. Patients from urban areas were 1.8 times (AOR = 1.80, 95% CI (1.12, 2.91)) more likely to have a good attitude than those who live in a rural area (Table 3).

**Table 3:** Factors affecting good attitude towards DM complication among Type 2 adult DM patients at Addis Zemen District Hospital, Gondar, Ethiopia, April 2019

Variables	Attitude status		COR(95%CI)	AOR(95%CI)
	Good attitude	Poor attitude		
Level of Education				
Can’t read and write	49	61	1	
Informal school and Can read and write	88	36	3.04(1.77,5.22)**	2.32(1.26, 4.27)**
Primary school and can read and write	60	17	4.39 (2.28,8.47)**	4.31 (2.06, 9.02)**
Secondary school and above	65	26	3.11 (1.73,5.62)**	2.79 (1.41, 5.50)**
Marital status				
Married	144	58	1	1
Divorced	25	12	0.84 (0.40,1.78)	0.96 (0.41, 2.22)
Widowed	22	27	0.33 (0.17,0.62)*	0.51 (0.24, 1.08)
Single	71	43	0.67 (0.41,1.08)	0.41 (0.22, 1.72)
Occupation				
Farmer	48	36	1	1
Government employee	29	17	1.25 (0.60, 2.62)	1.33 (0.58, 3.04)
Merchant	72	32	1.65 (0.91, 3.01)	1.56 (0.79, 3.06)
House wife	56	21	1.96 (1.01,3.79)*	2.18 (0.95, 4.49)
NGO worker	56	34	1.21 (0.66, 2.22)	1.12 (0.57, 2.19)
Residence				
Rural	87	72	1	
Urban	175	68		
Duration of DM (in years)			2.13 (1.40, 3.24)**	1.80 (1.12,2.91)**
1–5	186	84	1	
6–10	56	37	0.68 (0.41, 1.11)	0.79 (0.46, 1.38)
>=10	20	19	0.47 (0.24, 0.93)*	0.55 (0.25,1.25)
Income (ETB)				
<500	110	46	1	1
500–1500	36	32	0.47 (0.26,0.85)*	0.55 (0.28, 1.08)
1501–2500	50	36	0.58 (0.34, 1.01)*	0.52 (0.27, 1.99)
>2500	66	26	1.06 (0.60, 1.88)	0.69 (0.35,1.37)

*significant at p-value ≤ 0.05, ** significant at p-value ≤ 0.01

**Abbreviations:***ETB = Ethiopian Birr, DM = Diabetes mellitus, NGO = Non-governmental organizations, COR = Crude Odds Ratio, AOR = Adjusted Odds Ratio*

### Practice of type 2 diabetic patients towards complications of Diabetes

Almost half of the respondents (48.8% (95% CI: 44.0, 53.5)) had a good practice towards diabetic complications. More than two-thirds of the patients 280(69.7%) reported that they never forgot to take their medication. One hundred nine (27.1%) and 179(44.5%) respondents said that they stopped their medication when they felt better and felt worse, respectively. Only about 21(5%) respondents performed exercise daily. The majority (86.6%) of them change their diet according to the recommendations of their physician. Most (80.1%) of respondents monitor their blood glucose levels in the hospital. One hundred fifty-one (37.6%) of respondents monitor their blood glucose level monthly. Greater than 239 (59%) of respondents reported daily feet examination. Only 48(11.9%) of respondents use hot water to wash their foot and 45.5% take care when they cut their nails. Regular kidney and eye checkup was practiced by 29.9 and 7.7% of study participants, respectively. Nearly all (96.8%) of respondents do not smoke, and 188(46.8%) of the participants never drink alcohol (Table 4).

**Table 4:** Response distribution of practice on DM complications related questions among Type 2 adult DM patients at Addis Zemen District Hospital, Gondar, Ethiopia, April, 2019

Variables	Frequencies	%
Forget to take medicine/ insulin		
Yes	122	30.3
No	280	69.7
Careless at times about taking medicine		
Yes	114	28.4
No	288	71.6
Stop taking medicine when feel better		
Yes	109	27.1
No	293	72.9
Stop taking medicine when feel worse		
Yes	179	44.5
No	223	55.5
Physical work or exercise in the last week		
Never	186	46.3
Once a week	43	10.7
2–3 times per week	99	24.6
Almost daily	53	13.2
Daily	21	5.2
Duration of physical work or exercise		
< 10 Minutes/day	57	14.2
10-20 Minutes/day	74	18.4
20-30 Minutes/day	36	9.0
> 30 Minutes/day	49	12.2
Diet modification according to the recommendations of physician		
Yes	348	86.6
No	54	13.4
Monitoring blood glucose		
Self- monitoring	68	16.9
Local pharmacy	12	3.0
Hospital	322	80.1
Barriers for self-blood glucose monitoring		
Too expensive	128	31.8
Too painful	6	1.5
Don’t know how to test	77	19.2
Other*	123	30.6
Frequency of blood glucose monitoring		
Monthly	151	37.6
Every 2 month	165	41.0
Every 3 month	86	21.4
checking feet		
Never	41	10.2
Daily	239	59.5
Once a week	6	1.5
Rarely	116	28.9
Drinking alcohol		
Never	188	46.8
Weekly	34	8.5
2–3 times weekly	50	12.4
Nearly every day	29	7.2
Rarely	101	25.1
Cigarette smoking		
Yes	13	3.2
No	389	96.8
Wearing footwear during exercise as recommended by health professionals		
Yes	196	48.8
No	206	51.2
Type of water used to wash feet		
Cold water	354	88.1
Warm water but not hot	48	11.9
care during cutting toe nails		
Yes	183	45.5
No	219	54.5
Periodic kidney examination		
Yes	120	29.9
No	282	70.1
Regular checkup of eye by eye specialist		
Yes	31	7.7
No	371	92.3
Practice level		
Good practice	196	48.8
Poor practice	206	51.2

Abbreviations: DM = Diabetic mellitus *Incontinence, lack of motivation, frustration

### Factors associated with practice towards complications of Diabetes

Educational status was the single independent factor associated with patient practice towards diabetic complications. The odds of good practice towards prevention of complications of diabetes were two times (AOR = 1.96, 95% CI (1.06, 3.61)) more likely among type 2 diabetic patients with educational level of informal school and can read and write, four times (AOR = 4.31, 95% CI (2.06, 9.02)) more likely among participants who attained primary school and can read and write and two-point six times (AOR = 2.57, 95% CI (1.32, 5.02)) more likely among those who attained secondary school and above than those who cannot read and write (Table 5).

**Table 5:** Factors affecting good practice towards DM complications among Type 2 adult DM patients at Addis Zemen District Hospital, Gondar, Ethiopia; April, 2019

Variables		Practice level		COR(95%CI)	AOR(95%CI)
		Good practice	Poor practice		
**Age**		N			
18–30		81	53	1	1
31–45		53	65	0.53 (0.32,0.88)*	0.69 (0.35, 1.40)
	> = 45	62	88	0.46 (0.29,0.74)*	0.59 (0.30, 1.15)
**Level of education**					
Can’t write and read		33	77	1	1
Informal school and can Read and write		67	57	2.74 (1.60, 4.70)**	1.96 (1.06, 3.61) *
Primary school and can read and write		38	39	2.27 (1.24, 4.16)*	1.53 (0.78, 3.01)
Secondary school and above		58	33	4.10 (2.27, 7.40)**	2.57 (1.32, 5.02)**
**Marital status**					
Married		94	108	1	
Divorced		18	19	1.09 (0.54, 2.20)	1.13 (0.51, 2.52)
Widowed		16	33	0.56 (0.29, 1.08)	0.99 (0.46, 2.12)
Single		68	46	1.70 (1.07, 2.71)	1.11 (0.58, 2.13)
**Occupation**					
Farmer		38	47	1	
Government employee		12	34	0.44 (0.20,.96)	0.39 (0.17,1.90)
Merchant		59	45	1.62 (0.91,2.89)	1.49 (0.79, 2.82)
House wife		37	40	1.14 (0.62,2.12)	1.33 (0.68, 2.59)
NGO worker		50	40	1.55 (0.85,2.81)	1.41 (0.73, 2.72)
**Resident**					
Rural		63	96	1	
Urban		133	110	1.84 (1.23,2.77)*	1.49 (0.94, 2.37)
**Duration of DM**					
≤5		147	123	1	
6–10		36	57	0.53 (0.33,0.86)*	0.65 (0.38, 1.13)
≥10		13	26	0.42 (0.21,0.85)*	0.57 (0.26, 1.27)
**Level of income**					
< 500		80	75	1	
500–1500		28	40	0.67 (0.37, 1.18)	1.07 (0.55, 2.08)
1501–2500		33	53	0.59 (0.35, 1.01)	0.82 (0.43, 1.55)
> 2500		55	37	1.41 (0.84, 2.38)	1.71 (0.89, 3.25)

*significant at p-value ≤ 0.05, ** significant at p-value ≤ 0.01

Abbreviations: *DM = Diabetes mellitus, NGO = Non-governmental organizations, COR = Crude Odds Ratio, AOR = Adjusted Odds Ratio*

## Discussion

The present study discloses important information on the level of attitude and practice towards diabetes complications and its associated factors among type 2 DM patients in Ethiopia at Addis Zemen District Hospital. This study showed that over two-thirds (65.2%) of the study participants had a good attitude level while less than half (48.8%) of study participants had a good practice on DM complications. This finding is supported by a study done in Bangladesh, which reported that 66.4% of participants had a good attitude but regarding practice, only 20.1% were in good practice [20], which is lower than the finding of the present study. The justification may be because of socio-economic and cultural differences.

Most (79.9%) of the study participants in this study said that they could prevent diabetic complications. This finding is in line with the finding of the same study in Bangladesh where most study participants had reported that they could prevent diabetic complications [20], while other findings in Ethiopia, India, and Pakistan reported only 52.7, 55.6 and 62.3%, respectively [22, 29, 30]. In this study, good glycemic control was assumed to prevent diabetic complications by only 16.9% of respondents. On the contrary, the study done in Bangladesh showed that the majority (66.4%) of the study participants considered good glycemic control as one way to prevent it [20]. This variation could be due to a lack of awareness of type 2 diabetic patients about the effects of uncontrolled blood glucose levels. This may be because of a lack of counseling by health professionals about the effect of poor glycemic control, as it is the major cause of DM complications, because of their work overload.

Only 37.6% of respondents of the current study monitor their blood glucose level monthly, which is lower than the finding of a study in Bangladesh (95.8%) [20].

This difference could be because of a low awareness of patients on the importance of glycemic control to prevent diabetic complications since only 16.9% of respondents believe that good glycemic control is important to prevent diabetic complications.

Most (79.9%) participants of the current study believed that regular exercise prevents further complications. This is in agreement with the study conducted in Bangladesh (70%) [20], but less than the study in India 97.3% [31]. However, only 5% respondents in this study performed exercise daily, which is comparable with the study conducted in Pakistan (8.6%) [32]; but less than the study conducted in Oman (40%) [31], India (40.3%) [33], West Ethiopia (63.5%) [23], and Bangladesh (57%) [20]. This low activity in the current study might be because of a lack of habit of exercise or a lack of commitment to exercise by the study participants. It might also be because of the inappropriateness of the living environment to exercise.

Inconsistent with the study done in India (95.9%), dietary modification was considered a beneficial practice to prevent diabetic complications (91.8%) [31]. Regarding practice, 86.6% of participants practiced dietary modification which is higher than compared to previous studies in Oman [33], India [31], and west Ethiopia [23], which reported a 56, 49.7, and 69.5% of dietary modification practice, respectively. This higher value in Ethiopia as compared to other countries could be because of the content of the diet recommended by health professionals which is almost similar to what they are practicing regularly. In Ethiopia, the most commonly consumed food is in Amharic ‘injera [34]. Injera is composed primarily of teff, the smallest grain in the world which is grown primarily in Ethiopia. It is the best choice for helping to control blood glucose [35]. Over 59 % of respondents reported about daily feet examination. This finding is higher than the study done in Nigeria (40.9%) [36] and Bangladesh [20], where less than 50% of respondents reported about regular feet examination [20]. On the other hand, this finding is lower than the findings of the study done in Saudi Arabia (79.5%) [21] and Pakistan (94.4%) [37]. This variation could be due to a difference in culture and socioeconomic status.

Only 7.7% of the current study participants visited an eye specialist. The same finding is reported in Pakistan (10%) [32]. This could be due to lack of awareness about eye complications or due to the absence of an eye specialist at the diabetic center, since the research was conducted at district hospital which is known that there is a lack of specialists.

The current study revealed that 29.9% of the study participants have periodical kidney examinations which is lower than the finding of the study conducted in Bangladesh (64%) [20]. This might be due to the lack of a laboratory facility for real examination or a lack of awareness about diabetic complications.

The present study showed that educational status was significantly associated with a good attitude. This finding is supported by other studies [20, 38]. Subjects who can read and write, attended primary school, and secondary school and above were 2, 4.3, and 2.8 times more likely to have a good attitude than those who cannot read and write, respectively. Type 2 diabetic patients who were educated could have a higher chance of accessing and reading different materials like leaflets, manuals, and books and can communicate with health care professionals with no barrier. This helps them to gather information and improve their attitude towards DM complications.

The residence was also found to be significantly associated with a good attitude. Participants from urban areas were nearly two times more likely to have a good attitude than those who lived in rural areas. It is consistent with other studies [38]. This might be due to a difference in access to information. In urban areas there are different ways of accessing information like television, internet, and other technology Media which are not available in rural areas. Besides, in Ethiopia, health facilities are mostly located in urban areas, which creates a good opportunity for DM patients to have frequent visits and contact with health professionals. This provides an opportunity to gain more information regarding DM complications and to upgrade their attitude.

The level of education was found to be significantly related to practice. This is supported by other studies [18, 20, 23, 30, 39]. Patients who can read and write and high school and above were 2 and 2.6 times more likely to have a good practice to prevent diabetic complications than those who cannot read and write, respectively. This might be because educated participants can read necessary information easily from different written documents; this in turn helps them to improve their level of practice. As the level of education increases, the chance of attending different conferences and seminars on DM also increases. This helps patients to increase their awareness and to improve their practice.

## Limitations

Since the data about attitude and practice of DM complications were self-reported, there may be a recall bias and they may respond only to socially acceptable responses that might cause an overestimation of some results. The study was conducted on all type 2 diabetic patients without considering their diabetic complication history status during the data collection period which affects their attitude and practice level positively or negatively. Though patients who were severely ill were excluded, some patients having mild complications (e.g. Nephropathy) have been included in the study and such complications could have also affected their attitude and practice. Some questions used to assess attitude (diabetes spoils my social life, diabetes is the worst thing that happened to me) have some limitations on assessing attitude towards diabetes complications because it could be affected by patients opinions.

## Conclusions

A greater proportion of DM patients have a relatively good attitude but poor practice. Educational status was significantly associated with both good attitude and practices, whereas residence was a significant contributing factor for a good attitude only. The current study suggests the need for well-organized health educational programs and counseling on complications of diabetes regularly to improve their attitude and practice towards it.

## Supplementary information

**Additional file 1.**

## Data Availability

The data will be available upon request from the corresponding author.

## Abbreviations

DM: Diabetic Mellitus
NGO: Non-governmental Organization
AOR: Adjusted Odds Ratio
CI: Confidence Interval
COR: Crude Odds Ratio
SPSS: Statistical Package for Social Sciences
IDF: International Diabetic Federation
