# Supplementary material for: Attitude, practice and its associated factors towards Diabetes complications among type 2 diabetic patients at Addis Zemen District hospital, Northwest Ethiopia
Source: BMC Public Health. 2020 May 26;20:785. doi: 10.1186/s12889-020-08953-6 (PMC7249631; doi:10.1186/s12889-020-08953-6)
Supplement: Supplementary file 1 — Additional file 1. [file 12889_2020_8953_MOESM1_ESM.docx]

**Additional files:**

Data collection tools used for Attitude, Practice, and its Associated Factors towards Diabetes Complications among Type 2 Diabetic Patients at Addis Zemen District Hospital, Northwest Ethiopia, April, 2019.

1. ***Socio demographic characteristics***
2. *Sex*
   1. *Male*
   2. *Female*
3. *Age*
   1. *18-30*
   2. *31-45*
   3. *45-70*
4. *Level of Education*
   1. *Cannot read and write*
   2. *Informal education can read and write*
   3. *Primary school and can read and write*
   4. *Secondary school and above*
5. *Marital status*
   1. *Married*
   2. *Divorced*
   3. *Widowed*
   4. *Single*
6. *Occupation*
   1. *Farmer*
   2. *Government worker*
   3. *Merchant*
   4. *Housewife*
   5. *NGO worker*
7. *Religion*
   1. *Orthodox*
   2. *Muslim*
   3. *Protestant*
8. *Ethnicity*
   1. *Amhara*
   2. *Kimant*
   3. *Tigrie*
9. *Residence*
   1. *Rural*
   2. *Urban*
10. *Duration of DM*
    1. *(1-5)*
    2. *(6-10)*
    3. *>10*

*10. Type of medication they use*

- 1. *Oral*
  2. *Injectable*
  3. *Both*

1. *Family history of DM*
   1. *Yes*
   2. *No*
2. *Income( ETB)*
   1. *<500*
   2. *500-1500*
   3. *1501-2500*
   4. *>2500*
3. ***Attitude questions and their respective score***
4. *Do you think you can lead a normal life if you take appropriate measures for diabetes? Agree “1” neither agree and disagree and disagree “0”*
5. *Regular exercise prevents further complication? Agree “1”, neither agree and disagree and disagree”0”*
6. *Glycemic control has no role in preventing diabetic complications? Disagree “1”, while agree and neither agree and disagree given score of “0”*
7. *My diabetic diet spoils my social life? Agree and neither agree and dis agree scored as “0” but disagree scored as “1”*
8. *Do you believe that you could prevent diabetic complications? Agree “1” neither agree and disagree”0”*
9. *Do you agree that dietary modification is important to prevent diabetic complication? Agree “1” neither agree and disagree”0”*
10. *Do you agree Weight reduction is important to prevent diabetic complication? Agree “1” neither agree and disagree”0”*
11. *I avoid telling people I have diabetics? Agree and neither agree and dis agree scored as “0” but disagree scored as “1”*
12. *Diabetic is the worst thing that has ever happened to me? Agree and neither agree and dis agree scored as “0” but disagree scored as “1”*
13. ***Practice Questions***
14. *Do you ever forget to take your medicine/ insulin?*
15. *Are you careless at times about taking your medicine?*
16. *When you feel better do you sometimes stop taking your medicine?*
17. *Sometimes if you feel worse when you take the medicine, do you stop taking it?*
18. *How often have you done physical work or exercise in the last week?*
19. *How long do you exercise during physical work or exercise?*
20. *Do you modify your diet according to the recommendations of your physician?*
21. *How do you monitor your blood glucose?*
22. *What are barriers for self-blood glucose monitoring?*
23. *How frequent you monitor your blood glucose level.*
24. *How often you check your checking feet?*
25. *How often do you drink alcohol?*
26. *Do you smoke cigarette?*
27. *Will you wear footwear as recommended by your health worker when you go to exercise?*
28. *What type of water do you use to wash your feet?*
29. *Do you take care when you cut your toe nails?*
30. *Do you conduct periodic kidney examination?*
31. *Do you take regular checkup of eye by eye specialist?*
